# Clinical evolution of bladder carcinosarcoma: A case report and literature review

**DOI:** 10.1097/MD.0000000000039225

**Published:** 2024-08-09

**Authors:** Shuo Wu, Xiaolong Wang, Yuliang Zuo, Kuan Wang, Meihong Ye, Chaoming Wang

**Affiliations:** a Department of Urology, Huanghe Sanmenxia Hospital Affiliated to Henan University of Science and Technology, Sanmenxia, Henan Province, China; b Department of Pathology, Huanghe Sanmenxia Hospital Affiliated to Henan University of Science and Technology, Sanmenxia, Henan Province, China.

**Keywords:** bladder carcinosarcoma, clinical evolution, prognosis, transurethral resections of bladder tumors, TURBT

## Abstract

**Rationale:**

Bladder carcinosarcoma (BC) is a malignant tumor composed of a mixture of malignant epithelial and stromal components. Carcinosarcoma mostly occurs in the upper respiratory tract and upper gastrointestinal tract and is less common in the urinary system. The incidence of malignant tumors of the urinary system is <3%. It rarely occurs in the bladder and accounts for approximately 0.31% of all malignant bladder tumors. A literature review and this report will help to further improve our understanding, diagnosis, and treatment of bladder carcinosarcoma (BC).

**Patient concern:**

We describe the case of an 80-year-old female patient who was admitted to the hospital with a history of intermittent hematuria for 3 years. Furthermore, total cystectomy was refused when a BC was diagnosed. Palliative resection surgery was necessary because of the recurrent hematuria and abdominal pain.

**Diagnoses:**

Pathologically confirmed BC after surgery.

**Interventions:**

The patient’s first transurethral resection of bladder tumor (TURBT) was diagnosed as BC. However, the patient refused a total cystectomy. Two months after intravesical treatment with epirubicin, bladder tumor recurrence was observed during follow-up cystoscopy. The patient underwent a second TURBT for hemostatic treatment due to persistent hematuria. Due to hematuria and abdominal pain, a third TURBT was performed to reduce tumor size and stop bleeding. Finally, tumor recurrence resulted in bilateral hydronephrosis, and the patient underwent bilateral renal catheter drainage guided by B-ultrasound.

**Outcomes:**

Bladder carcinosarcoma caused uremia, electrolyte imbalance, and sepsis. Approximately 19 months after the discovery of the tumor, the patient died.

**Lessons:**

Radical bladder resection is recommended once a BC is diagnosed. By reporting the cases and reviewing the literature in the database, we will summarize the epidemiology, origin, etiology, clinical features, existing treatments, and prognostic factors of BC, and propose new prospects for BC therapy.

## 1. Introduction

Bladder carcinosarcoma is a rare malignant neoplasm characterized by a compact admixture of malignant epithelial components (carcinomas) and mesenchymal components (sarcomas).^[1]^ Compared with bladder urothelial carcinoma, carcinosarcoma has a later stage and worse prognosis. One-year overall survival rates for patients with bladder urothelial carcinoma and carcinosarcoma were 77% and 48%, respectively, and the 5-year overall survival rates were 47% and 17%, respectively.^[2]^ According to previous research, radical cystectomy and/or multimodal treatment (chemotherapy or radiotherapy) significantly reduces the risk of death compared to bladder preservation surgery.^[3,4]^ However, guidelines related to this disease are limited because of the rare incidence and poor prognosis of bladder carcinosarcoma (BC).

Here, we report the evolution, diagnosis, treatment, and poor prognosis of BC. Combining our experience with previous reports, we reviewed BCs to gain a deeper understanding of this disease.

## 2. Case report

**Figure 1.**
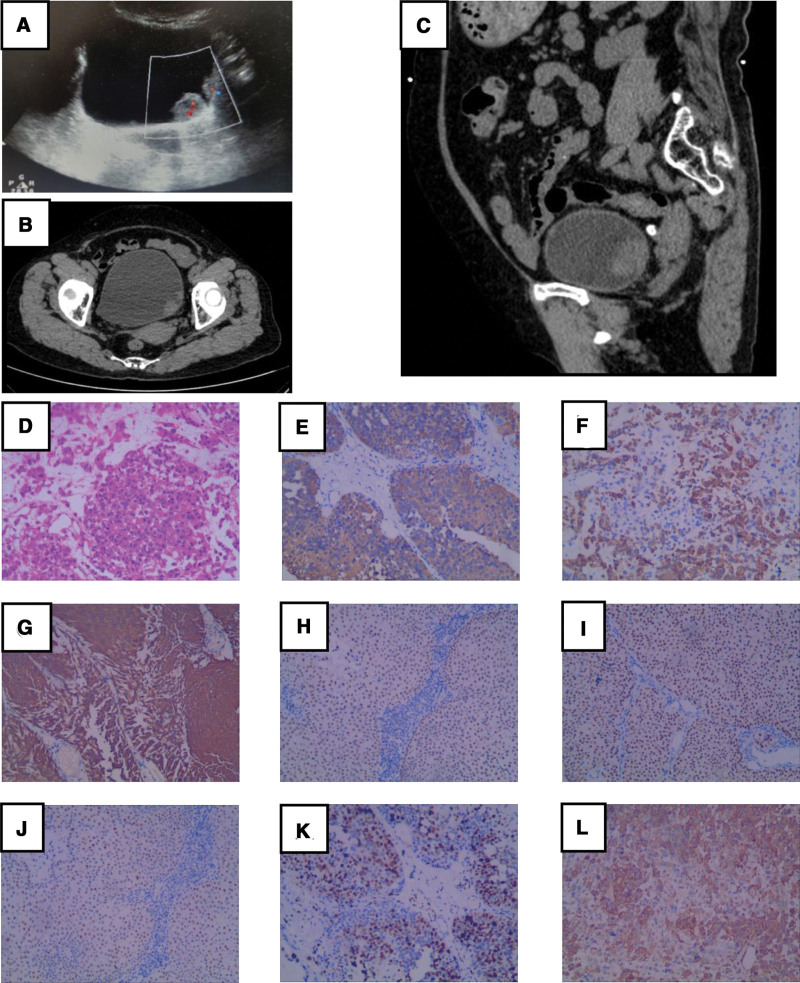
(A) Ultrasound examination reveals 2 masses on the left wall of the bladder. (B, C) Computed tomography examination shows a mass on the left side of the bladder (horizontal and sagittal planes). (D) Hematoxylin and eosin staining indicated that the tumor was malignant bladder carcinosarcoma. Tumor cells are (E) CK20 (+), (F) CK5/6 (+), (G) CK7 (+), (H) GATA3 (+), (I) P40 (+), (J) P53 (+), (K) Ki-67 (+70%), (L) SMA (+). Magnification, 100×.

On August 1, 2020, an 80-year-old female was transferred to our hospital because of gross hematuria with coagulation clot discharge for 3 days, accompanied by frequent urination, urgency, pain during urination, and increased nocturia (7–8 times at night). She had a history of repeated intermittent hematuria for more than 3 years without diagnosis and treatment. The patient had a history of diabetes for 10 years, whose blood sugar control. Urinary system color ultrasound (Fig. 1A) and computed tomography (CT) (Fig. 1B and C) showed that the left side wall of the bladder was occupied by 2 lumps (26.8 × 16.7 cm, 14.7 × 10.9 cm). On August 12, 2020, transurethral resection of a bladder tumor (TURBT) was performed. During the surgery, we found that the tumor had invaded and blocked the left ureteral orifice. Therefore, a ureteral D-J tube was placed to prevent ureteral stenosis. Postoperative pathology confirmed that the patient had a BC (Fig. 1D). Immunohistochemical analysis revealed the following: CK20 (+), CK5/6 (+), CK7 (+), GATA3 (+), P40 (+), P53 (+), Ki-67 (+70%), and SMA (+) (Fig. 1E–L). Pathological results showed that the tumor has not invaded the muscular layer. According to the TNM system and clinical staging of bladder cancer by the American Joint Committee on Cancer,^[5]^ the pathological stage is T1N0M0 and the clinical stage is stage I. After reviewing the latest literature and guidelines, we informed the patients and their families about the prognosis and latest diagnosis and treatment opinions of BC and strongly recommended radical cystectomy. The patient refused radical cystectomy, radiation therapy, or intravenous chemotherapy; therefore, we administered a regular bladder infusion of pirarubicin at 40 mg/week.

**Figure 2.**
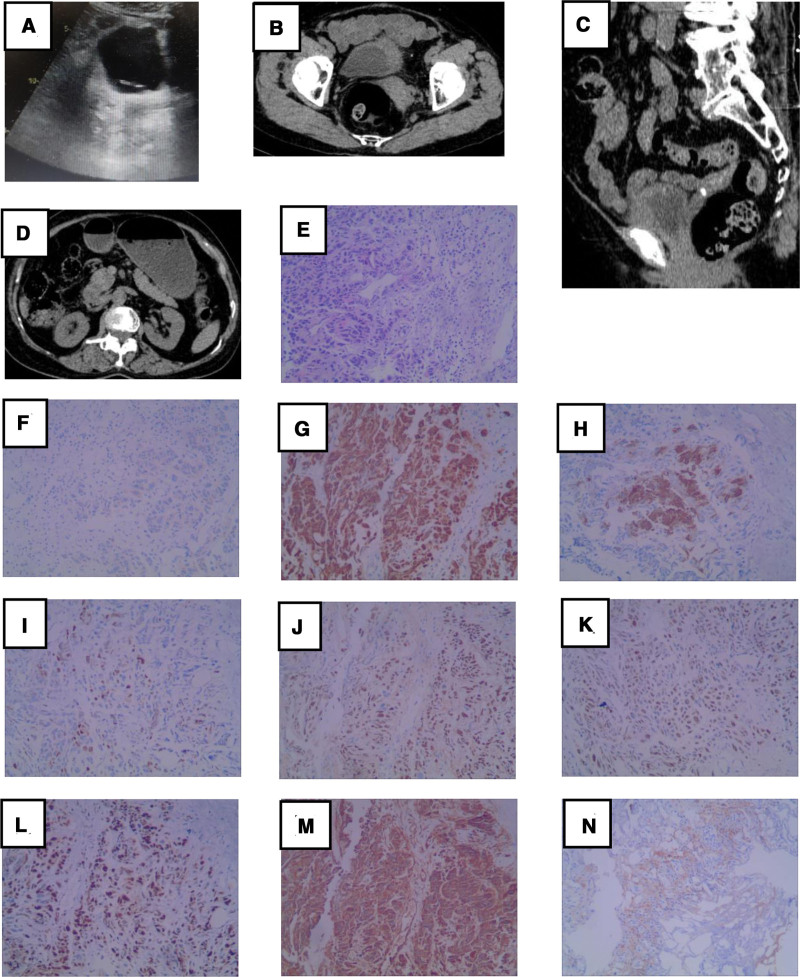
(A) Ultrasound reveals ureteral D-J tube placed during first surgery. (B, C) Single-photon emission computed tomography (SPECT) reveals tumor recurrence in the anterior and left wall of the bladder (horizontal and sagittal planes). (D) SPECT shows no hydronephrosis. (E) The second postoperative pathological tissue staining with hematoxylin and eosin confirmed bladder carcinosarcoma. Tumor cells are (F) CK20 (−), (G) CK5/6 (+), (H) CK7 (scattered+), (I) GATA3 (Scattered+), (J) P40 (+), (K) P53 (+), (L) Ki-67 (+30%), (M) SMA (+), (N) PD-L1 (+tumor cells 1%, +immune cells 3%). Magnification, 100×.

The ureteral D-J tube was removed on October 20, 2020 (Fig. 2A). Cystoscopy revealed a tumor measuring approximately 1 cm on the left wall of the bladder, indicating tumor recurrence. The patient refused a radical cystectomy. Simultaneously, the patient discontinued intravesical treatment.

On December 23, 2020, the patient was readmitted to the hospital because of gross hematuria. Single-photon emission computed tomography (SPECT) showed tumor recurrence on the anterior and left side walls of the bladder, with the thickest part approximately 1.7 cm (Fig. 2B and C), no hydronephrosis (Fig. 2D), and no bone or lymphatic metastasis. During the second TURBT, a protruding mass measuring approximately 2.0 × 3.0 cm in size was seen on the left side of the bladder, which did not involve the bilateral ureteral orifice. The pathological findings were consistent with those of previously diagnosed BC (Fig. 2E). Immunohistochemical analysis revealed the following: CK20 (−), CK5/6 (+), CK7 (scattered+), GATA3 (scattered+), P40 (+), P53 (+), Ki-67 (+30%), SMA (+), and programmed death protein 1 (PD-L1) (clone number: 142) (+tumor cells, 1%; +immune cells, 3%). (Fig. 2F–N). The pathological results were still non-muscle-invasive tumors, and the pathological and clinical stages were the same as before.

**Figure 3.**
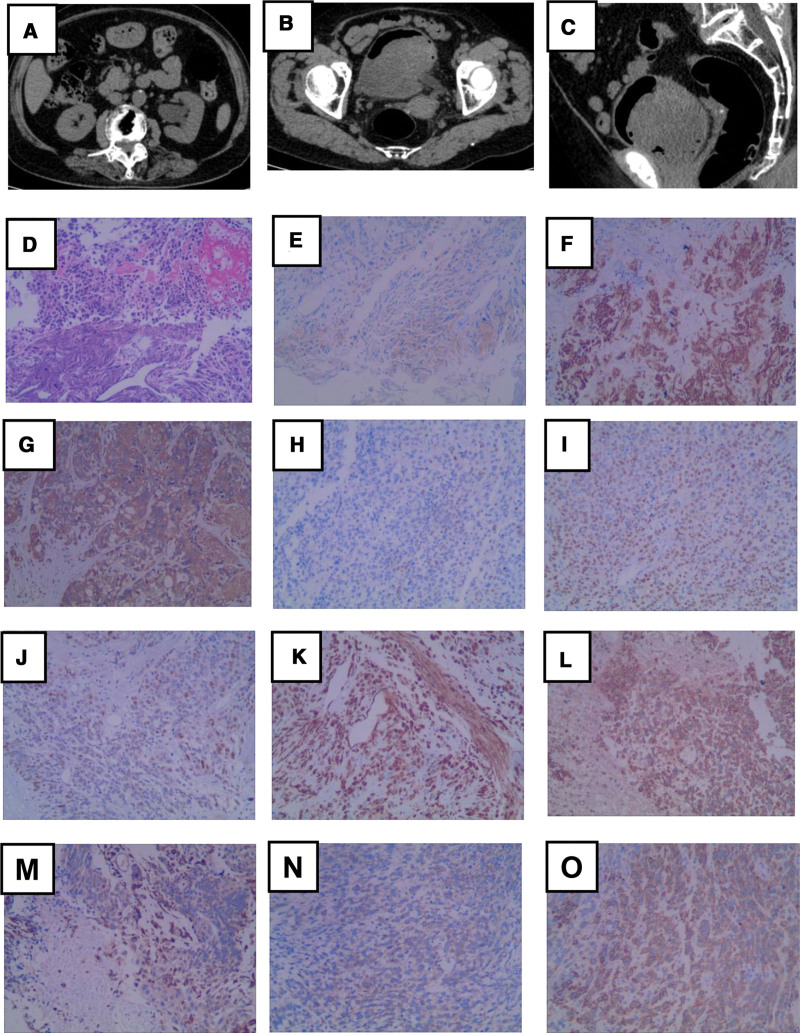
(A) Computed tomography (CT) shows left hydronephrosis. (B, C) CT shows a huge tumorous mass about 8 × 8 cm in the bladder (horizontal and sagittal planes). (D) Hematoxylin and eosin staining confirmed bladder carcinosarcoma. Tumor cells are (E) CK20 (+), (F) CK5/6 (local+), (G) CK7 (+), (H) GATA3 (scattered+), (I) P40 (+), (J) P53 (scattered+), (K) Ki-67 (+80%), (L) SMA (+), (M) vimentin (+), (N) MyoD1 (+), (O) CK (+). Magnification, 100×.

On June 30, 2021, she was readmitted to the hospital because of hematuria and abdominal pain. Routine blood examination revealed anemia. Computed tomography revealed left hydronephrosis (Fig. 3A) and postoperative recurrence of the BC (Fig. 3B and C). TURBT was performed for the third time to reduce bleeding and alleviate pain. We performed a 2-hour operation to remove a large carcinosarcoma-like solid mass (8 × 8 cm) in the bladder; however, the left ureteral opening could not be found. The patient’s discomfort was significantly relieved postoperatively. The patient was satisfied with the surgery results. Pathology revealed BC with high-grade urothelial carcinoma as the cancer component, accompanied by large necrosis (Fig. 3D). Immunohistochemical analysis revealed the following: CK20 (+), CK5/6 (local+), CK7 (+), GATA3 (scattered+), P40 (+), P53 (scattered+), Ki-67 (+80%), SMA (+), vimentin (+), MyoD1 (+), and CK (+). (Fig. 3E–O). Pathological results confirmed that is the muscle-invasive tumor, which in a stage of at least T2. But accurate staging could not be confirmed because the patient refused further examination.

**Figure 4.**
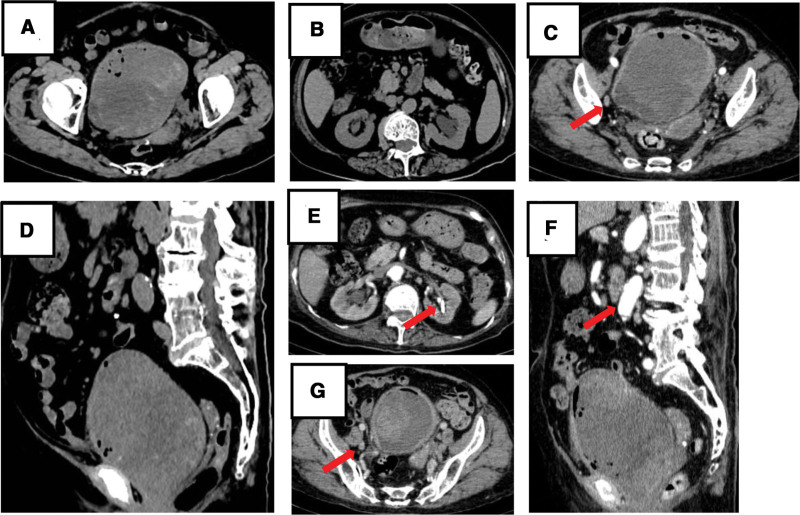
(A, B, and D) show computed tomography (CT) scans (November 17, 2021). (C, E, F, and G) show single-photon emission computed tomography (SPECT) scans (November 25, 2021). (A, D) CT images showing the tumor-filling bladder (Horizontal and sagittal planes). (B) Computed tomography (CT) showing hydronephrosis in the right and left kidneys. (C, G) SPECT showing tumor recurrence and metastasis to the lymph nodes. (Red arrow indicates the right pelvic lymph node.) (E) SPECT image showing bilateral hydronephrosis. (Red arrow indicates the nephrostomy tube previously placed under B-ultrasound guidance) (F) Sagittal view of SPECT. (Red arrows indicate enhancing vessels to confirm that this is a contrast-enhanced scan.)

A follow-up CT scan 4 months later showed that the BC had recurred, filled the entire bladder (Fig. 4A and D), and was accompanied by bilateral hydronephrosis (Fig. 4B and E). We performed bilateral percutaneous nephrostomy drainage under B-ultrasound guidance. SPECT revealed neoplasm recurrence and metastasis to the right pelvic lymph nodes (Fig. 4C, G, and F). On February 28, 2022, the patient was readmitted to the hospital due to tumor cachexia. Blood tests were performed, and the results are presented in (Table 1). Eventually, the patient died 1 week after hospitalization due to uremia, electrolyte imbalance, and sepsis. No autopsies were performed.

**Table 1:** Patient’s blood test results on February 28, 2022.

Test item	Detection value	Reference value
Leukocyte	75.49 × 10^9^/L	(3.5–9.5) × 10^9^/L
Neutrophil percentage	98%	(40–75) %
Neutrophil count	73.9 × 10^9^/L	(1.8–6.3) × 10^9^/L
Red blood cells	3.08 × 10^12^/L	(3.8–5.1) × 10^12^/L
HB	80 g/L	(150–150) g/L
K^+^	7.09 mmol/L	(3.5–5.3) mmol/L
Na^+^	112 mmol/L	(137–147) mmol/L
Bun	28.17 mmol/L	(2.86–8.2) mmol/L
Cr	707 μmol/L	(41–81) μmol/L
Albumin	25.5 g/L	(40–55) g/L
PCT	5.02 ng/mL	(0–0.5) ng/mL
CRP	223.5 mg/L	(0–8) mg/L
Urine culture	*Escherichia coli*	–

– = not detected, Bun = blood urea nitrogen, Cr = serum creatinine, CRP = c-reactive protein, HB = hemoglobin, PCT = procalcitonin.

## 3. Discussion

In 1887, Shattock reported the first case of a BC.^[1]^ WHO Health Organization defines carcinosarcoma as a malignant tumor consisting of a mixture of malignant epithelial and mesenchymal constituents, which is infrequent in clinical practice. Other terms used for carcinosarcoma include spindle cells, metaplastic, and sarcomatoid carcinomas.^[6]^ Urothelial carcinoma is a common bladder cancer; however, carcinosarcoma is rare, accounting for only 0.3% of all bladder malignancies.^[7]^ Carcinosarcomas typically have a higher T stage and more active regional and distant metastases when discovered and carry a higher risk of death than urothelial carcinomas.^[2]^ Wang et al^[8]^ considered that tumor stage was the only factor affecting the prognosis of BC. Liu et al^[9]^ reported that BC is associated with poor differentiation, advanced stage, and poor overall survival, and cancer-specific survival.

The histological origin of carcinosarcoma has not yet been determined, and there are mainly the following theories: collision tumor theory, stromal induction theory, polyclonal theory, and monoclonal theory.^[10]^ By analyzing loss of heterozygosity and X-chromosome inactivation, Sung et al obtained consistent nonrandom X-chromosome inactivation and the overlap of loss of heterozygosity between the carcinomatous and sarcomatous components, which supports the notion of monoclonal origin of the 2 components of carcinosarcoma.^[11]^ Similarly, other investigators have found that the epithelial and mesenchymal components of BC share identical TP53 mutation patterns and nuclear p53 immunohistochemical staining characteristics, suggesting a common clonal origin.^[12]^ Research by Halachmi et al^[13]^ suggests that both the cancerous and sarcomatous components of carcinosarcoma originate from a common stem cell. Downregulation of E-cadherin may block the transformation of epithelial cells to a sarcoma phenotype. The epithelial component may consist of squamous cell carcinoma, carcinoma in situ, adenocarcinoma, transitional cell carcinoma (TCC), and small cell carcinoma.^[1,14]^ The component of sarcomas include rhabdomyosarcoma, leiomyosarcoma, osteosarcoma, chondrosarcoma, liposarcoma, and malignant fibrous histiocytoma.^[1,7,10]^

However, the etiology of carcinosarcoma remains unclear. Cyclophosphamide is well regarded as a contributory factor of cause for urothelial carcinoma of the urinary bladder. The patient who received long-term cyclophosphamide treatment developed a carcinosarcoma of the urinary bladder.^[15]^ Some researchers believe that long-term intravesical instillation of pirarubicin may lead to transformation of urothelial carcinoma into carcinosarcoma.^[16]^ There is a report that local schistosomiasis infection can lead to BC.^[17]^ Risk factors for BC have been reported in the literature, such as chronic inflammation of the urinary system, bladder diverticula, Bacillus Calmette–Guérin (BCG) intravesical instillation, long-term indwelling catheterization, bladder stones, smoking, male sex, and race.^[7]^

Malla et al^[18]^ summarized the clinical symptoms of 835 patients with BC from 15 reports, and found that the clinical symptoms of patients with BC were analogous to those of patients with bladder urothelial carcinoma. Hematuria and dysuria are usually the main symptoms, which is consistent with the case in our report, in which hematuria was the first symptom. Bladder carcinosarcoma tumors are usually 1.5 to 13 cm in size. It usually occurs on the side wall and bottom of the bladder, and is rarely observed in the trigone of the urinary bladder. Carcinosarcoma appears as dark gray, pedunculated, polypoid, or broad-based intraluminal masses that often invade the bladder muscle layer.^[18]^ But when the tumor invades the ureteral orifice, it is worth considering whether to put a ureteral D-J tube during the first diagnostic resection in our case. Does this increase the risk of tumor metastasis? When a large bladder mass and rapid clinical progression are observed, BC should be considered. However, the final diagnosis of carcinosarcoma needs to be confirmed by pathology and immunohistochemistry.

At least 1 of the 2 immune markers (cytokeratin 20 and p53) is aberrantly expressed in urothelial carcinoma in situ (CIS). In urothelial CIS, cytokeratin 20 (CK20) and/or p53 usually increase and CD44 levels decrease.^[19]^ In an immunohistochemical study of epithelial tumors, cytokeratin 5/6 (CK 5/6) immunoreactivity was detected in 15 of the 24 transitional cell carcinomas (62%).^[20]^ Cytokeratin 7 (CK7) expression is positive in most primary bladder cancers.^[21]^ Although p53 has relatively low sensitivity (30%) in urothelial CIS, it has a specificity of 100% in CIS, with strong and diffusely positive nuclear reactivity.^[22]^ The expression of GATA3 is correlated with poor prognosis of patients, positively correlated with tumor cell division, and negatively correlated with overall patient survival.^[23]^ One study demonstrated the utility of uroplakin II and ΔNp63 (p40) in the diagnosis of primary bladder urothelial carcinoma, and that uroplakin II and GATA3 may be useful in the diagnosis of urothelial carcinoma metastasis. High-intensity p40 immunostaining (3+) is inversely associated with patient survival.^[24]^ Immunohistochemistry revealed abnormal expression of α-smooth muscle actin (SMA) in leiomyosarcoma,^[25]^ rhabdomyosarcoma,^[26]^ angiomyolipoma,^[27]^ and other sarcoma tissues. Immunohistochemical markers showed differential responses to different components of sarcoma. For example, desmin was positively expressed in rhabdomyosarcoma.^[28]^ And muscle-specific actin (MSA) is positively expressed in leiomyosarcoma.^[29]^ Vimentin and cytokeratin antibodies were used in combination to identify carcinosarcoma and synovial sarcoma, respectively.^[30]^ Positive expression of myogenin and/or MyoD1 is helpful in the diagnosis of spindle cells or sclerosing rhabdomyosarcoma.^[31]^ Carcinosarcoma was considered if the tumor expressed both the carcinomatous and sarcomatous components. Immunohistochemistry results showed that the expression of the cancer epithelial component markers was CK20 (+), CK5/6 (+), CK7 (+), GATA3 (+), P40 (+), CK (+), and P53 (+). In the present case, the mesenchymal tissue was positive for SMA, vimentin, and MyoD1 expression. Myogenin expression was negative (the immunohistochemical image of myogenin-negative expression is shown in Supplemental Fig. 1, Supplemental Digital Content, http://links.lww.com/MD/N315). The sarcomatous component of BC that we report may consist of leiomyosarcoma, rhabdomyosarcoma, or various sarcomatous tissues. The positive expression of PD-L1 in tumor and immune cells suggests that PD-L1 immunosuppressants may be beneficial for the treatment of BC.

The treatment for carcinosarcoma remains controversial and is currently under investigation. Bladder carcinosarcoma has a poor prognosis with only 18.4-month median overall survival. Radical cystectomy alone or in combination with multimodal treatment (radiotherapy or chemotherapy) markedly decreases mortality compared to bladder-sparing surgery alone. There was no difference in survival between the groups that underwent radical cystectomy alone and those that received neoadjuvant or adjuvant chemotherapy.^[3]^ Oppositely, very few researchers have reported that the prognosis of carcinosarcoma of the bladder is no worse than that of high-grade urothelial carcinoma. They did not differ significantly in terms of grade, stage, rate of positive surgical margins, lymph node involvement, incidence of associated prostate cancer or progression, or all-cause or disease-specific mortality. The most important prognostic factor is stage, not histology.^[32]^ And there is a report of pathological complete response of BC with gemcitabine and carboplatin combined with outpatient radiotherapy,^[33]^ which may be an effective treatment modality for BC. Based on our case, we believe that if the patient refuses total cystectomy, TURBT followed by chemotherapy, radiotherapy, or intravesical instillation is beneficial in reducing recurrence. SPECT revealed only pelvic lymph node metastasis a few months before the patient’s death. If a patient undergoes early radical resection for BC, the prognosis may improve.

In addition to conventional surgery, radiotherapy, and chemotherapy, new immunosuppressive agents have recently been used in the clinical treatment of malignant tumors. A series of programmed death protein 1/programmed death-ligand 1 (PD-1/PD-L1) inhibitors (avelumab, atezolizumab, durvalumab, nivolumab, and pembrolizumab) have been approved for use in patients with locally advanced or metastatic urothelial carcinoma by the Food and Drug Administration.^[34]^ When PD-1 and PD-L1 block the PD-1/PD-L1/2 signaling pathway, T lymphocytes are activated to kill tumor cells.^[35]^ Up to date, there have been no reports on the therapeutic effects of immunotherapy in BC. Immunohistochemistry revealed PD-L1 positivity in the present case. A thought-provoking question is whether the application of PD-L1 inhibitors prolongs the survival time and improves the quality of life of patients. The first BC cell line, Mannheim sarcoma (MaS-3), was successfully established and reported in 2021.^[36]^ MaS-3 showed carcinosarcoma characteristics consistent with those of the original tumor in immunocytochemistry, genomic analysis, and proteomics. In addition, the authors verified that the MaS-3 cell line was highly sensitive to gemcitabine, 5-fluorouracil, and cisplatin in chemical sensitivity.^[36]^ Undoubtedly, the MSA-3 cell line will help researchers understand and elucidate the characteristics and treatment of this rare carcinoma. In the future, researchers could use the MAS-3 cell line to conduct various experiments to verify whether PD-1/PD-L1 is effective in the treatment of carcinosarcoma and to identify new therapeutic schedules for patients with BC.

## 4. Conclusion

The evolution of our cases confirmed that BC is extremely malignant. Even if TURBTs is performed by skilled surgical experts, the recurrence and metastasis rates remain high. Therefore, radical cystectomy is strongly recommended for patients with early-stage BC.

## Author contributions

**Data curation:** Shuo Wu, Xiaolong Wang, Kuan Wang.

**Investigation:** Shuo Wu, Xiaolong Wang, Kuan Wang.

**Visualization:** Shuo Wu, Meihong Ye.

**Writing – original draft:** Shuo Wu.

**Writing – review & editing:** Yuliang Zuo, Chaoming Wang.

**Conceptualization:** Chaoming Wang.

## Supplementary Material
