# Supplementary figures and images for: Clinical evolution of bladder carcinosarcoma: A case report and literature review
Source: Medicine (Baltimore). 2024 Aug 9;103(32):e39225. doi: 10.1097/MD.0000000000039225 (PMC11315510; doi:10.1097/MD.0000000000039225)

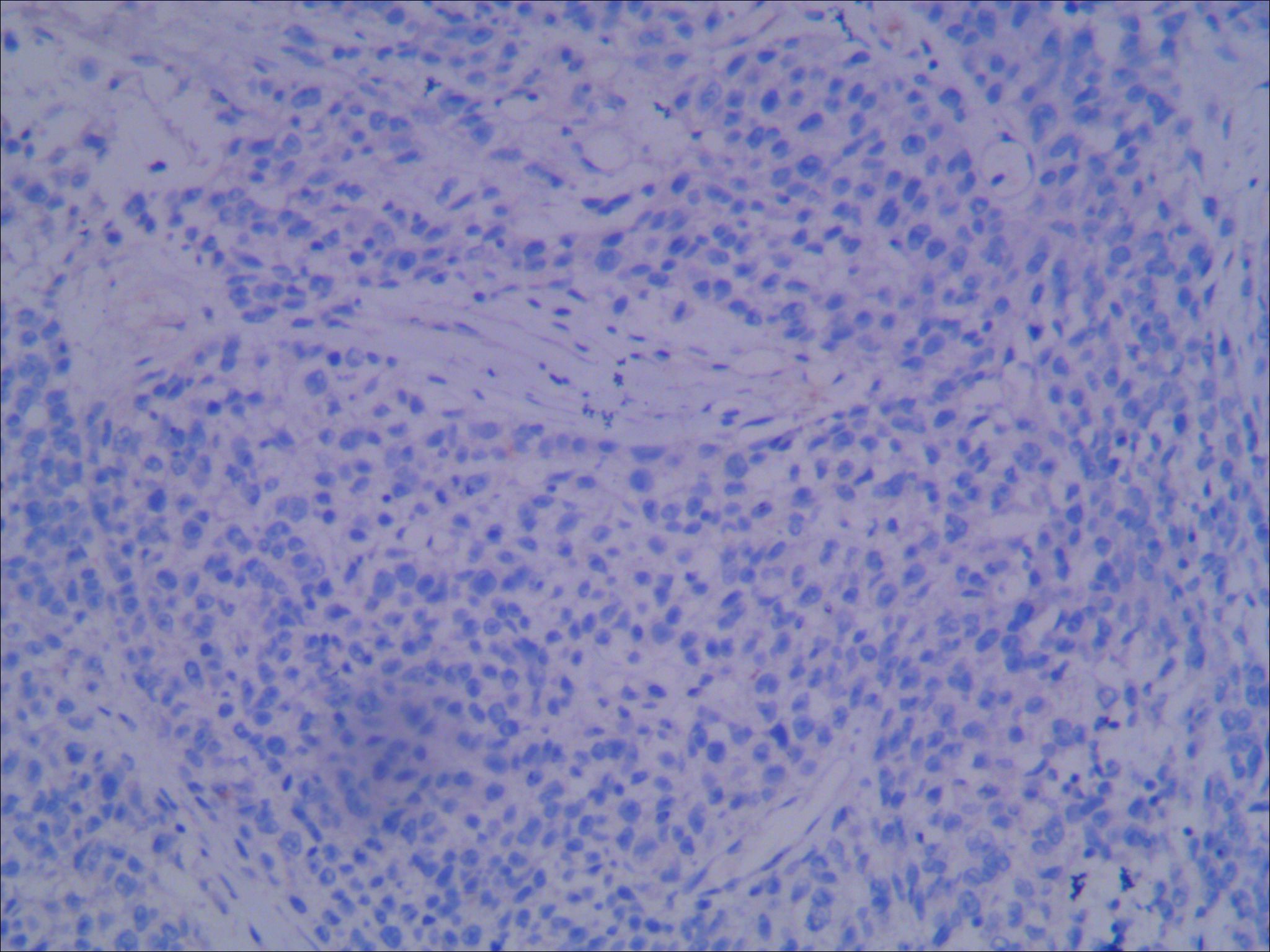

Supplement: Supplementary file 1 [file medi-103-e39225-s001.jpg]
